# Pharmacological Characterization of Vasomotor Responses in the Tree Shrew (*Tupaia belangeri*) Basilar Artery: A Promising Model for Human Cerebrovascular Research

**DOI:** 10.3390/biology15141146

**Published:** 2026-07-14

**Authors:** Md. Zahorul Islam, Mohammad Enamul Hoque Kayesh, Michinori Kohara, Kyoko Tsukiyama-Kohara, Atsushi Miyamoto

**Affiliations:** 1 Department of Veterinary Pharmacology, Joint Faculty of Veterinary Medicine, Kagoshima University, 1-21-24 Korimoto, Kagoshima 890-0065, Japan; khokonpharma@gmail.com; 2 Department of Pharmacology, Faculty of Veterinary Science, Bangladesh Agricultural University, Mymensingh 2202, Bangladesh; 3 Transboundary Animal Diseases Centre, Joint Faculty of Veterinary Medicine, Kagoshima University, 1-21-24 Korimoto, Kagoshima 890-0065, Japan; mehkayesh@pstu.ac.bd (M.E.H.K.); kkohara@vet.kagoshima-u.ac.jp (K.T.-K.); 4 Laboratory of Animal Hygiene, Joint Faculty of Veterinary Medicine, Kagoshima University, 1-21-24 Korimoto, Kagoshima 890-0065, Japan; 5 Department of Microbiology and Public Health, Faculty of Animal Science and Veterinary Medicine, Patuakhali Science and Technology University, Barishal 8210, Bangladesh; 6 Department of Microbiology and Cell Biology, Tokyo Metropolitan Institute of Medical Science, 2-1-6 Kamikitazawa, Setagaya-ku, Tokyo 156-8506, Japan; kohara-mc@igakuken.or.jp

**Keywords:** *Tupaia belangeri*, cerebral artery, receptor, cerebrovascular regulation, animal model

## Abstract

The tree shrew (*Tupaia belangeri*) is increasingly recognized as an important experimental model for studying human diseases due to its close phylogenetic relationship with humans. Because the basilar artery’s response to endogenous vasoactive mediators varies among species, we investigated the effects of vasoactive substances on isolated tupaia basilar arteries to determine whether this species is suitable as a human model. 5-Hydroxytryptamine (5-HT) and histamine (His) induced contraction, while bradykinin (BK) and acetylcholine (ACh) produced concentration-dependent relaxation. Pharmacological analysis revealed that contractions triggered by 5-HT were regulated through 5-H_1_ and 5-HT_2_ receptors, while His-induced responses were mediated by H_1_ receptors. BK-induced relaxation was inhibited by the B_2_ antagonist HOE140 and *N^ω^*-nitro-L-arginine (L-NA), but not by B_1_ antagonists or indomethacin, suggesting a B_2_ receptor-mediated nitric oxide (NO) pathway. ACh-induced relaxation was markedly reduced in the presence of L-NA and the M_3_ antagonist pFHHSiD, indicating M_3_ receptor-mediated NO release. At basal tone, L-NA evoked a contractile response, while indomethacin led to relaxation, suggesting basal regulation by NO and prostanoids. These findings demonstrate that 5-HT_1_/5-HT_2_ and H_1_ receptors mediate contraction, while B_2_ and M_3_ receptors mediate relaxation in the tupaia basilar artery. This study highlights species-specific cerebrovascular regulation and supports the tupaia as a relevant model for investigating human cerebrovascular physiology and pathophysiology.

## Simple Summary

We pharmacologically characterized the basilar artery of the tree shrew (*Tupaia belangeri*) using several endogenous vasoactive substances. The artery showed strong contractions to 5-hydroxytryptamine and histamine, relaxations to bradykinin and acetylcholine that were mediated by nitric oxide, very weak contraction to noradrenaline, and no response to angiotensin II in vitro. Overall, most response patterns resembled those reported for human cerebral arteries, indicating that the tree shrew may serve as a valuable model for cerebral vascular diseases. These results also emphasize the importance of species-specific vascular characteristics when interpreting results, and further in vivo and molecular studies are needed.

## 1. Introduction

Animal models are commonly employed to study complex biological processes and to replicate key features of human diseases [1,2]. Among these models, non-human primates are particularly valuable because they share a close genetic relationship with humans and can therefore simulate human pathology and physiology with high fidelity [3]. Nevertheless, their high cost and low reproductive capacity substantially limit their utility. The tree shrew (*Tupaia belangeri*), commonly called tupaia, a small diurnal mammal in the family Tupaiidae, has gained increasing attention as a biomedical research model due to its close evolutionary relationship to primates [4,5,6,7]. In contrast to rodents, tree shrews exhibit greater genetic, anatomical, physiological, and neuroanatomical similarity to humans, notably including a primate-like, relatively large brain-to-body ratio [4,5,6,8,9]. In addition, they are readily maintained under laboratory conditions owing to their small size, ease of breeding, short gestation, rapid maturation, low maintenance cost, and straightforward handling [10]. These characteristics have enabled their use across diverse fields, including viral hepatitis, oncology, metabolic disease, aging, psychiatric disorders, myopia, preclinical drug evaluation, and cognitive neuroscience, making them a versatile and promising complementary model for human cerebrovascular research and comparative physiology [7,11,12,13,14].

The basilar artery supplies blood to the brainstem, cerebellum, and posterior cerebral cortex [15]. Its tone is finely regulated by various vasoactive substances, including acetylcholine (ACh), 5-hydroxytryptamine (5-HT), noradrenaline (NA), bradykinin (BK), angiotensin II (Ang II), and histamine (His) [16,17]. These mediators are essential for maintaining cerebral homeostasis but are also critically implicated in the pathophysiology of cerebrovascular diseases such as subarachnoid hemorrhage, stroke, hypertension, and vascular dementia. Cerebral arterial function is often markedly impaired after circulatory disturbances, and blunted responses to vasoactive agents may arise primarily from endothelial or vascular smooth muscle dysfunction [18,19]. To elucidate mechanisms of cerebral circulatory disorders and inform prevention and treatment strategies, multiple animal models have been established.

Vascular responsiveness to endogenous agents shows pronounced interspecies variability. For example, NA elicits contraction in dogs [20] and monkeys [21] but induces relaxation in cattle [22] and pigs [23], whereas BK causes vasodilation in humans but contraction in horses [24] and bats [25]. These divergent responses complicate extrapolation to human physiology and underscore the need for appropriate models in cerebrovascular research. Our previous work has systematically compared basilar artery reactivity across vertebrate taxa, including mammals (e.g., equine, bovine, porcine, deer, dolphins, bats, rodents), birds (chickens), and reptiles (snakes), supporting the hypothesis that vascular responsiveness correlates with the evolutionary complexity of the brain [23,24,25,26,27,28,29,30,31]. Based on this evidence, we hypothesize that, with increasing brain evolutionary complexity, the basilar artery responds to a broader range of vasoactive substances.

Despite the promise of the tree shrew as a model organism, its cerebrovascular physiology remains largely uncharacterized. To address this critical gap, the present study assesses the vasomotor responses of the tupaia basilar artery to six vasoactive agents—NA, 5-HT, Ang II, BK, His, and ACh. These mediators act through diverse receptor pathways and can elicit vasoconstriction, vasodilation, or mixed effects. However, their specific influence on tree shrew cerebral vessels has not been defined. By characterizing basilar artery reactivity in vitro, this work aims to elucidate mechanisms of vascular regulation in this species, provide critical comparative insights into interspecies variability, and establish the tree shrew as a promising model for translational cerebrovascular research.

## 2. Materials and Methods

### 2.1. Tissue Preparation

**Figure 1:**
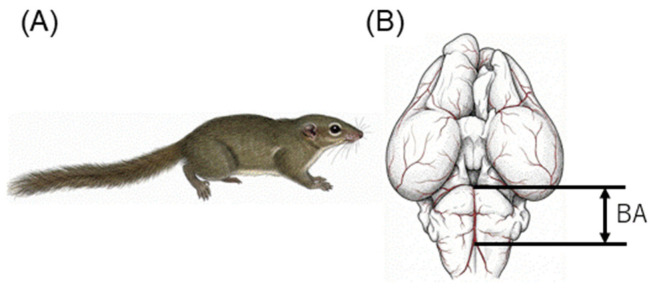
(**A**) A *Tupaia belangeri*. (**B**) A basilar artery (BA) on the medulla oblongata, dorsal view.

We received decapitated heads of tupaias (*n* = 18; body weight: 150.3 ± 6.7 g; age: 6–12 months; both sexes) from the Transboundary Animal Diseases Centre, Joint Faculty of Veterinary Medicine, Kagoshima University, Japan, where the animals were kept separately in individual cages and provided with eggs, fruit, water, and dry mouse food. All animal-handling procedures followed the laboratory animal care standards approved by the Institutional Animal Care and Use Committee (IACUC). Tupaias were anesthetized through intramuscular administration of ketamine at 5 mg/kg and atropine at 0.25 mg/kg, while anesthesia was sustained using 0.2% isoflurane (Fujifilm Wako Co. Osaka, Japan). After anesthesia and decapitation, the heads were provided to our laboratory. The basilar arteries (Figure 1) were carefully removed from the brain and placed in ice-cold physiological saline solution containing 119 mM NaCl, 4.7 mM KCl, 1.6 mM CaCl_2_, 1.2 mM MgCl_2_, 25 mM NaHCO_3_, 1.2 mM KH_2_PO_4_, and 10 mM glucose. The solution was maintained at pH 7.4 and continuously oxygenated with carbogen gas consisting of 95% O_2_ and 5% CO_2_. Under a stereomicroscope, surrounding connective tissues were carefully removed from each artery immediately after isolation. The study procedures were also carried out in compliance with the ARRIVE guidelines and were approved by the Ethics Committee of Kagoshima University (Approval No. VM1217, date of approval: 12 November 2012).

### 2.2. Reagents

The chemicals used in this study, along with their final bath concentrations, were as follows:

Noradrenaline (NA; 10^−9^–10^−5^ M), histamine hydrochloride (His; 10^−8^–10^−4^ M), diphenhydramine hydrochloride (10^−5^ M), cimetidine (10^−5^ M), angiotensin II acetate salt (Ang II; 10^−9^–10^−5^ M), bradykinin acetate salt (BK; 10^−10^–10^−6^ M), des-Arg^9^-(Leu^8^)-BK (10^−5^ M), methoctramine hydrate (10^−6^ M), *N^ω^*-nitro-L-arginine (L-NA; 10^−4^ M), and sodium nitroprusside (SNP; 10^−4^ M), all obtained from Sigma-Aldrich, St. Louis, MO, USA. 5-Hydroxytryptamine (5-HT; 10^−9^–10^−5^ M) was purchased from Merck, Darmstadt, Germany. HOE140 (10^−6^ M) was supplied by Peptide Institute, Osaka, Japan, while indomethacin (10^−5^ M) was obtained from Nacalai tesque, Kyoto, Japan. Acetylcholine chloride (ACh; 10^−9^–10^−5^ M) was sourced from Daiichi Sankyo, Tokyo, Japan. Pirenzepine dihydrochloride (10^−6^ M) was purchased from Santa Cruz Biotechnology, Santa Cruz, CA, USA, and hexahydro-sila-difenidol hydrochloride, p-fluoro analog (pFHHSiD, 10^−6^ M) was obtained from Research Biochemicals, Natick, MA, USA. U-46619 (10^−7^ M) was supplied by Cayman Chemical Company, Ann Arbor, MI, USA. Distilled water was used as the solvent for preparing all reagent solutions.

### 2.3. Functional Studies

From each tupaia basilar artery, two arterial segments of about 2 mm in length were prepared. We used independent vessel segments for each set of experiments. The segments were positioned horizontally between two L-shaped stainless-steel wires with an external diameter of 0.05 mm. One wire was attached to an isometric force transducer (TB-611T, Nihon Kohden Kogyo, Tokyo, Japan), and the preparation was placed in a 4 mL water-jacketed microtissue organ bath (UMTB-1, Unique Medical Co., Ltd., Tokyo, Japan). The bath contained oxygenated physiological salt solution maintained at 37 °C and pH 7.4. After mounting, each arterial ring was allowed to stabilize for a minimum of 120 min under a resting tension of 0.1 g. This preload was used because it produced the strongest contractile response in the basilar artery. To assess tissue viability and obtain stable contraction, 60 mM KCl was applied at 30 min intervals until the contractile response reached a constant value. During the equilibration period, the preparations were washed repeatedly with fresh salt solution more than ten times. The NaCl level in the physiological solution was adjusted equimolarly to compensate for changes in KCl concentration. Isometric force signals were amplified using an AP-621G amplifier (Nihon Kohden Kogyo, Tokyo, Japan), converted into digital data through a PowerLab/8SP analog-to-digital converter (ADInstruments Co., Castle Hill, NSW, Australia), and recorded on a computer. A cumulative concentration–response curve was generated by adding increasing concentrations of each agonist directly into the organ bath. The concentration ranges of the vasoactive agents were selected according to our earlier studies [22,25,26,27]. When antagonists were used, they were applied to the bath solution 30 min before agonist treatment. None of the antagonists altered the basal tone of the vessels.

### 2.4. Statistical Analysis

Data are presented as means ± SEM. Concentration–response curves were analyzed using two-way ANOVA followed by Bonferroni’s multiple-comparison post hoc test. Statistical analyses were performed using GraphPad Prism version 9.4.1 (GraphPad Software, San Diego, CA, USA). A value of *p* < 0.05 was considered statistically significant.

## 3. Results

### 3.1. Responsiveness to 5-HT, His, NA, Ang II, BK, and ACh

**Figure 2:**
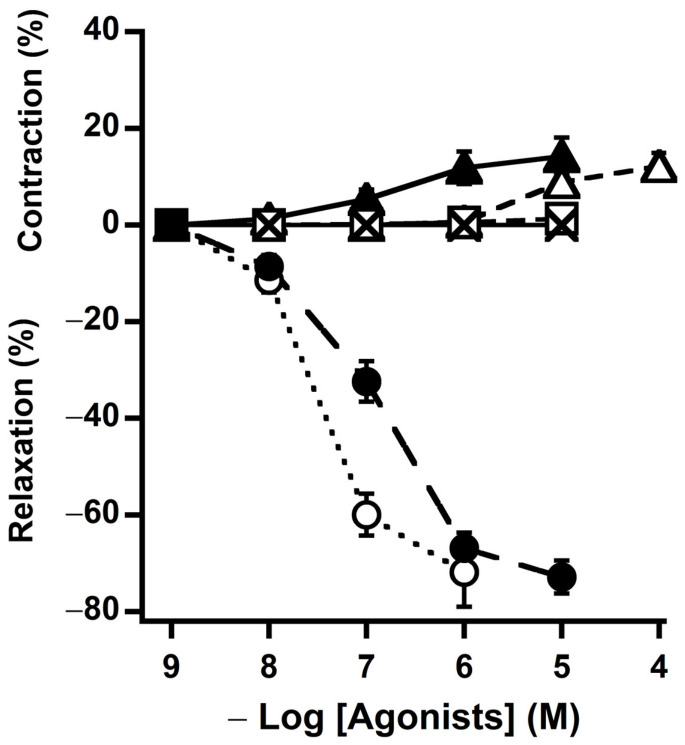
Effect of 5-hydroxytryptamine (▲), histamine (△), noradrenaline (□), angiotensin II (×), bradykinin (○), and acetylcholine (●) on isolated tupaia basilar arteries. Vascular contraction was determined under basal resting tension and reported as a percentage of the contraction elicited by 60 mM KCl. For relaxation studies, arteries were precontracted with U-46619 (10^−7^ M), after which responses to bradykinin and acetylcholine were measured and expressed relative to the relaxation produced by sodium nitroprusside (10^−4^ M). All values are shown as mean ± SEM from 5 to 6 tupaias.

Concentration-dependent vascular responses to 5-HT, His, NA, Ang II, BK, and ACh were examined in isolated basilar arteries from Tupaia (Figure 2). Contractile effects were recorded from arteries maintained at their basal resting tone. In contrast, relaxation was assessed after the vessels had been pre-constricted (about 40–50% of 60 mM KCl) with U-46619, a thromboxane A_2_ analogue, at 10^−7^ M, following confirmation that the tested agents did not produce contraction under resting conditions. We used U-46619 for preconstriction because it produced a stable and reproducible contraction (45.9 ± 3.7% of 60 mM KCl). Among the tested compounds, 5-HT, NA, and His produced concentration-related contractions, while ACh and BK caused concentration-dependent relaxations (Figure 2). The peak contractile effects of 5-HT, NA, and His were expressed as percentages of the response induced by 60 mM KCl. Similarly, the maximal relaxant effects of ACh and BK were calculated relative to the relaxation produced by 10^−4^ M SNP. For each vasoactive agent, pEC_50_ values, defined as the negative logarithm of the concentration required to elicit 50% of the maximal response, were also calculated and are presented in Table 1. Ang II had no vasomotor effects on the tupaia basilar artery. Among the agents tested, 5-HT induced the most potent contraction (14.2 ± 3.8%), whereas ACh was the most sensitive relaxing agent (pEC_50_ = 7.68 ± 0.13) and induced the strongest relaxation (–72.8 ± 3.4%) of the tupaia basilar artery.

### 3.2. Responsiveness to L-NA and Indomethacin

The influence of nitric oxide (NO) and prostaglandin pathways on basal tone in the tupaia basilar artery was examined using L-NA, a NO synthase inhibitor, and indomethacin, a cyclooxygenase inhibitor. Under resting conditions, L-NA at 10^−4^ M produced a contractile response equivalent to 8.32 ± 0.7% of the response to 60 mM KCl. In vessels precontracted with L-NA, indomethacin at 10^−5^ M caused relaxation amounting to 28.3 ± 3.1% of the relaxation produced by 10^−4^ M SNP.

### 3.3. Effect of 5-HT on the Isolated Tupaia Basilar Artery

**Figure 3:**
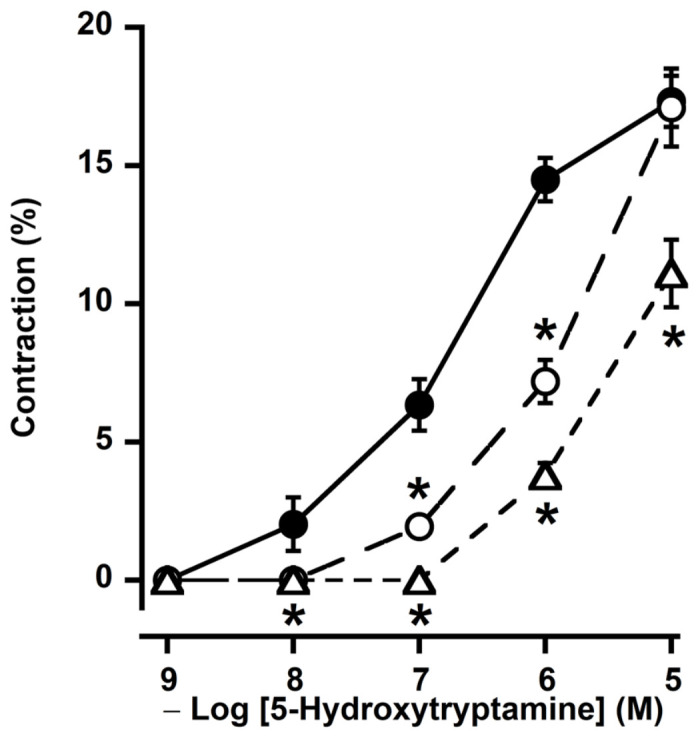
Influence of ketanserin (○, 10^−6^ M), a 5-HT_2_ receptor antagonist, and methiothepin (△, 10^−6^ M), a 5-HT_1_/5-HT_2_ receptor antagonist, on contraction produced by 5-hydroxytryptamine (●) in isolated basilar arteries from tupaias. Data are presented as means ± SEM from three tupaias. An asterisk (*) indicates a significant difference at *p* < 0.05 from the contraction response to 5-hydroxytryptamine in the absence of receptor antagonists.

Application of 5-HT elicited concentration-dependent contraction in isolated Tupaia basilar artery preparations (Figure 3). The involvement of serotonergic receptors was assessed using ketanserin, a 5-HT_2_ receptor antagonist, and methiothepin, which blocks both 5-HT_1_ and 5-HT_2_ receptors. Ketanserin at 10^−6^ M produced a parallel displacement of the 5-HT concentration–response relationship to the right (Figure 3). Methiothepin at 10^−6^ M also reduced the sensitivity of the tissue to 5-HT, as shown by a rightward shift in the concentration–response curve (Figure 3).

### 3.4. Effects of Diphenhydramine and Cimetidine on His-Induced Contraction

**Figure 4:**
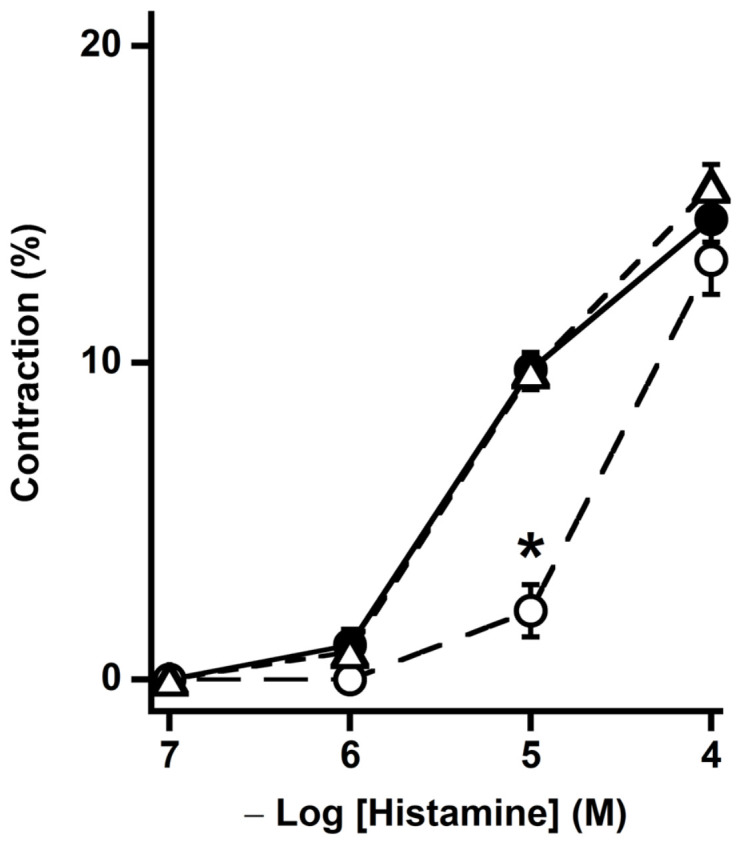
Influence of histamine receptor antagonists on contractile responses in isolated tupaia basilar arteries. The H_1_ receptor antagonist diphenhydramine (○, 10^−5^ M) and the H_2_ receptor antagonist cimetidine (△, 10^−5^ M) were tested against histamine-evoked contraction (●). Data are presented as means ± SEM from three tupaias. An asterisk (*) indicates a significant difference at *p* < 0.05 compared with the contraction produced by histamine alone, without antagonist treatment.

To determine the receptor subtype involved in the His-induced response, diphenhydramine (10^−5^ M; H_1_ receptor antagonist) and cimetidine (10^−5^ M; H_2_ receptor antagonist) were tested against the His concentration–response curve. Diphenhydramine produced a parallel displacement of the curve toward higher His concentrations, while cimetidine showed no statistically meaningful effect (Figure 4).

### 3.5. Effect of NA and Ang II on Isolated Tupaia Basilar Artery

NA induced a very weak contraction of isolated tupaia basilar arteries (1.34 ± 1.0%), preventing characterization of receptor involvement using antagonists. Ang II produced no vasomotor effects on the isolated tupaia basilar arteries.

### 3.6. Effects of L-NA, Indomethacin, and B_1_ and B_2_ Receptor Antagonists on BK-Induced Relaxation

**Figure 5:**
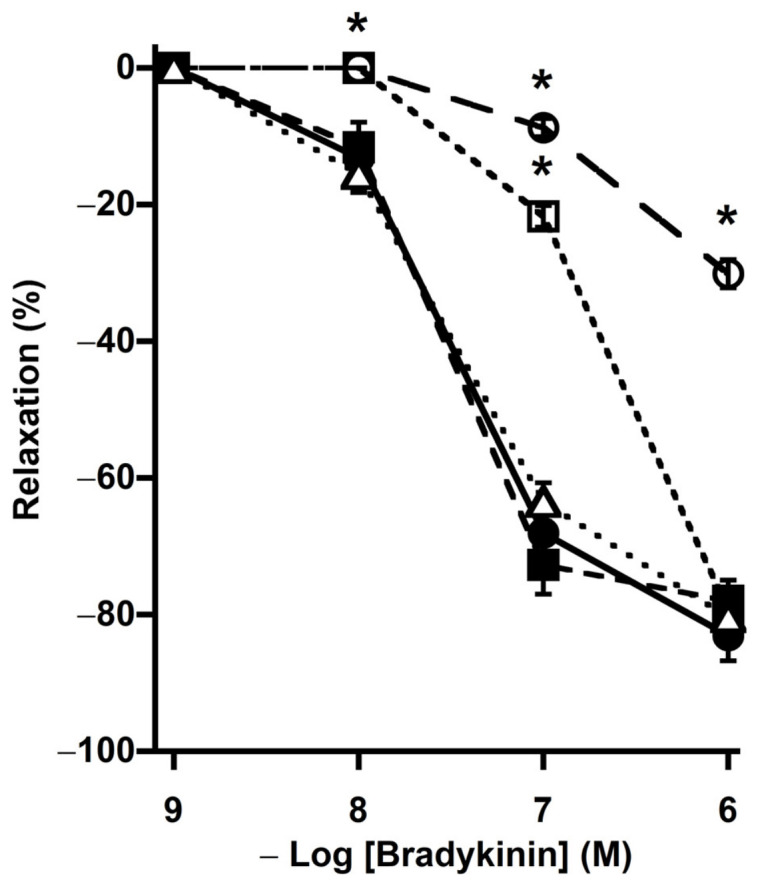
The influence of several pharmacological inhibitors on bradykinin-mediated relaxation was examined in isolated basilar arteries from tupaias. The tested agents included *N^ω^*-nitro-L-arginine (○, 10^−4^ M), an inhibitor of nitric oxide synthase; indomethacin (■, 10^−5^ M), a cyclooxygenase inhibitor; the B_1_ receptor antagonist des-Arg^9^-(Leu^8^)-BK (△, 10^−5^ M); and the B_2_ receptor antagonist HOE140 (□, 10^−6^ M). Bradykinin-induced relaxation (●) was measured after the arterial rings had been pre-contracted with U-46619 at 10^−7^ M. The relaxation was calculated as a percentage of the response produced by 10^−4^ M sodium nitroprusside. Data are presented as means ± SEM from 3 to 4 tupaias. An asterisk (*) indicates a significant difference at *p* < 0.05 compared with the relaxation caused by bradykinin alone, without any antagonist.

Treatment with L-NA, an inhibitor of NO synthase, markedly reduced the relaxation response produced by BK. In contrast, indomethacin, a cyclooxygenase inhibitor, did not produce any notable change in BK-mediated relaxation, as shown in Figure 5. To identify the receptor subtype involved in this response, the basilar arteries were exposed to selective antagonists for BK B_1_ and B_2_ receptors. Pretreatment with des-Arg^9^-(Leu^8^)-BK, a B_1_ receptor antagonist, at a concentration of 10^−5^ M did not significantly modify the relaxation caused by BK in tupaia basilar arteries. However, HOE140, which acts as a B_2_ receptor antagonist, shifted the BK concentration–response curve to the right in a parallel manner, indicating involvement of the B_2_ receptor subtype (Figure 5).

### 3.7. Effect of L-NA, Atropine, Pirenzepine, Methoctramine, and pFHHSiD on ACh-Induced Relaxation

**Figure 6:**
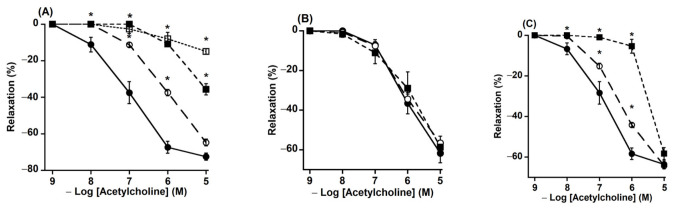
The influence of L-NA (□, 10^−4^ M) and atropine, the non-selective muscarinic antagonist (○, 10^−7^ M; ■, 10^−5^ M) on acetylcholine-mediated relaxation (●) was examined in isolated basilar arteries of the tupaia (**A**). The effects of selective muscarinic receptor antagonists were also evaluated: pirenzepine, an M_1_ receptor antagonist (○, 10^−6^ M), and methoctramine, an M_2_ receptor antagonist (■, 10^−6^ M), on acetylcholine-induced relaxation (●). (**B**). In addition, the role of M_3_ receptors was tested using pFHHSiD (○, 10^−7^ M; ■, 10^−5^ M) (**C**). The relaxation produced by acetylcholine was measured in arteries previously contracted with U-46619 at 10^−7^ M. The responses were calculated as a percentage of the relaxation caused by sodium nitroprusside at 10^−4^ M. Data are presented as means ± SEM from 3 to 4 tupaias. An asterisk (*) indicates a significant difference at *p* < 0.05 compared with acetylcholine-induced relaxation without antagonist treatment.

The role of NO synthesis and muscarinic receptor subtypes in ACh-mediated responses was examined using L-NA, atropine, pirenzepine, methoctramine, and pFHHSiD. L-NA markedly inhibited the relaxation produced by ACh, indicating that this response was largely dependent on NO. The non-selective muscarinic antagonist atropine at 10^−7^ M caused a rightward displacement of the ACh concentration–response curve, and this displacement became more pronounced at 10^−5^ M (Figure 6A). In contrast, blockade of M_1_ receptors with pirenzepine or M_2_ receptors with methoctramine did not produce a significant change in the ACh response (Figure 6B). However, inhibition of M_3_ receptors with pFHHSiD produced a concentration-dependent rightward shift in the ACh concentration–response curve (Figure 6C).

## 4. Discussion

This study presents the first pharmacological evaluation of the basilar artery of *Tupaia belangeri* in response to several endogenous vasoactive substances. 5-HT and His produced concentration-dependent contractions through activation of 5-HT_1_ and 5-HT_2_ and H_1_ receptors, respectively, whereas BK and ACh evoked potent endothelium-dependent, NO-mediated relaxation via activation of B_2_ and M_3_ receptors, respectively.

5-HT plays a significant role in regulating vascular tone within the intracranial circulation [32,33]. In most animal species, 5-HT-induced contraction has been reported to range from 40% to 100% [34]; however, in the tupaia basilar artery, we observed a maximum contraction of only 14.2%. This markedly lower contractile response may represent a distinctive functional characteristic of the tupaia basilar artery. It produced a dose-related contraction in the *tupaia* basilar artery. This response was antagonized by ketanserin, a 5-HT_2_ antagonist, and methiothepin, which blocks both 5-HT_1_ and 5-HT_2_ receptors. These findings suggest that both 5-HT_1_ and 5-HT_2_ receptor subtypes contribute to the contraction. This dual contribution is consistent with reports from various mammalian basilar arteries, where mixed 5-HT receptor subtype involvement is common. The contractile effects of 5-HT are mediated through activation of the 5-HT_1_ and 5-HT_2_ receptor subtypes in humans [35,36], dogs and monkeys (*Macaca fascicularis*) [37], and chickens [28]. These results suggest that serotonergic modulation is an important vasoregulatory pathway in the tupaia’s posterior circulation. These data highlight its potential significance in tupaia, particularly under pathological conditions such as subarachnoid hemorrhage or migraine, where local 5-HT levels are elevated. In contrast, NA induced only a weak contraction; therefore, detailed receptor characterization was not performed. A similarly attenuated response to adrenergic stimulation has been documented in human cerebral arteries [38], whereas in mice, NA produced no effect [29]. This suggests that adrenergic signaling plays a minor role in tupaia basilar artery compared with serotonergic pathways.

His produced concentration-dependent contraction that was selectively attenuated by the H_1_ antagonist diphenhydramine but not by the H_2_ antagonist cimetidine, implicating H_1_ receptors as the primary mediator of His-evoked contraction in the *tupaia* basilar artery. His-induced contraction of resting vascular tone has also been observed in several species, including humans [39], mice [29], and guinea pigs [40]. The H_1_ predominance suggests that histaminergic signaling in the *tupaia* basilar artery is potentially pro-contractile and may have pathophysiological relevance during allergic or inflammatory states when His release is elevated. His causes either vasoconstriction or vasodilation in human cerebral arteries due to the activation of H_1_ and H_2_-receptors, respectively [41,42]. The brain’s histaminergic pathway has a significant influence on cognitive processes, including learning and memory, and its dysfunction may be linked to neurological disorders that contribute to neurodegeneration [43,44,45]. Deficient histaminergic transmission in the human brain in vascular dementia has been suggested [45].

BK produced marked relaxation in pre-contracted tupaia basilar arteries in a concentration-dependent manner. This response was inhibited by the selective B_2_ receptor antagonist HOE140, whereas the B_1_ receptor antagonist des-Arg^9^-(Leu^8^)-BK had no effect. Inhibition of NO synthase (L-NA) substantially attenuated the BK response. Together, these data indicate a B_2_ receptor and NO-mediated relaxation induced by BK in the *tupaia* basilar artery. B_1_ receptor–mediated responses are typically not evident under normal physiological conditions [46]. BK causes relaxation of the human basilar artery through the activation of B_2_ receptors and depends on the release of an endothelial factor [47]. Endothelial B_2_ receptor activation has been shown to produce relaxation in human [47] and mouse [29] cerebral arteries; however, in porcine cerebral arteries, the same receptor activation has been associated with contraction [26]. BK-induced relaxation has also been reported in human forearm resistance vessels [48]. The strong B_2_-mediated relaxation in *tupaia* suggests that BK may act as a vasorelaxant in the posterior cerebral circulation of this species and could influence cerebrovascular responses during inflammation or ischemia.

ACh produced relaxation of pre-contracted *tupaia* basilar artery rings that were essentially abolished by L-NA, indicating that NO is the principal mediator of ACh-induced relaxation. Pharmacological blockade with muscarinic receptor antagonists showed that a clear M_3_-selective antagonist, pFHHSiD, shifted the concentration–response curve, while M_1_ and M_2_ antagonists had no effect. These results are in agreement with previous studies on chicken and mouse basilar arteries, which reported that atropine, a non-selective muscarinic receptor antagonist, and pFHHSiD, an M_3_ receptor antagonist, caused a rightward displacement of the ACh concentration–response curve. In contrast, the selective M_1_ and M_2_ receptor antagonists, pirenzepine and methoctramine, respectively, did not produce a comparable shift [28,29]. ACh-induced relaxation has also been reported in human cerebral artery [36]. Tupaia basilar artery may have rich cholinergic innervation similar to humans [49], which could contribute to a strong relaxation. These findings are consistent with classical endothelium-dependent muscarinic signaling, where M_3_ receptors on endothelial cells stimulate endothelial NO synthase.

NA induced only a minimal contraction in the *tupaia* basilar artery. This weak response is consistent with findings in the mouse basilar artery [29], which may be explained by the correlation between NA-induced vascular responsiveness and the gravitational load experienced by different species [31].

Ang II produced no detectable effect in the isolated basilar artery, suggesting either an absence or low expression of contractile AT_1_ receptors in this vascular bed of *tupaia*. This observation highlights species-specific patterns and points to caution when extrapolating renin–angiotensin system findings from other species to tupaia. Ang II induces contraction of human cerebral artery [49].

Blockade of basal NO production with L-NA produced a measurable contraction of resting arterial rings, whereas indomethacin produced relaxation under the same conditions. These opposing effects imply that, at rest, *tupaia* basilar artery tone is regulated by a balance between constitutive endothelial NO release and vasoconstrictor prostanoid activity. This tonic interplay is common among mammalian cerebral vessels and supports the presence of active endothelial control of basilar artery tone in *tupaia*.

Although the functional findings suggest the involvement of specific receptor pathways, direct confirmation of receptor expression was not performed. Future studies should therefore employ immunohistochemistry, qPCR, or complementary molecular techniques to confirm the expression and localization of 5-HT, His, BK, and muscarinic receptor subtypes in the tupaia basilar artery. In this study, the number of animals was kept to a minimum because of the limited availability of tupaias. In vivo experiments using tupaias would help to validate the physiological relevance of the present findings.

## 5. Conclusions

The basilar artery of *Tupaia belangeri* exhibits a distinctive vasomotor phenotype characterized by prominent 5-HT_1_ and 5-HT_2_ and H_1_-mediated contractile responses, B_2_-dependent BK action, potent M_3_/NO-mediated relaxation to ACh, weak adrenergic responsiveness, and little or no effect of Ang II in vitro. The characteristic cerebral vascular responses in tupaia, with the exception of the response to Ang II, were similar to those of humans among the mammalian species studied to date. These species-specific characteristics highlight the importance of carefully considering *tupaia* vascular biology when using this species as a model for human cerebrovascular research. Further in vivo and molecular studies are warranted to confirm these findings and to explore their relevance to human cerebrovascular disease.

## Figures and Tables

**Table 1:** The pEC_50_ values and maximal response (Max) to agonists.

Agonists	pEC_50_	Max (%)
Histamine	–	12.2 ± 2.7 ^a^
Noradrenaline	–	1.3 ± 1.0 ^a^
5-Hydroxytriptamine	–	14.2 ± 3.8 ^a^
Angiotensin II	–	No response
Bradykinin	7.07 ± 0.2	–71.9 ± 7.1 ^b^
Acetylcholine	7.68 ± 0.13	–72.8 ± 3.4 ^b^

^a^ Contractile response produced by 60 mM KCl was considered the reference value and expressed as 100%. ^b^ Relaxation produced by sodium nitroprusside at 10^−4^ M was also expressed as 100%. Histamine, noradrenaline, 5-hydroxytryptamine and angiotensin II produced either weak or no response; therefore, pEC_50_ values are not calculated. Data are presented as means ± SEM, with each value obtained from 5 to 6 tupaias.

## Data Availability

All data included in this article are available from the corresponding author.

## Abbreviations

The following abbreviations are used in this manuscript:

5-HT	5-Hydroxytryptamine
His	Histamine
BK	Bradykinin
ACh	Acetylcholin
Ang II	Angiotensin II
L-NA	*N^ω^*-nitro-L-arginine
NA	Noradrenaline
NO	Nitric oxide
SNP	Sodium nitroprusside
IACUC	Institutional Animal Care and Use Committee
pFHHSiD	Hexahydro-sila-difenidol hydrochloride, p-fluoro analog
